# Co-Selection of Bacterial Metal and Antibiotic Resistance in Soil Laboratory Microcosms

**DOI:** 10.3390/antibiotics12040772

**Published:** 2023-04-18

**Authors:** Ali Heydari, Nick D. Kim, Patrick J. Biggs, Jacqui Horswell, Gerty J. H. P. Gielen, Alma Siggins, Matthew D. Taylor, Collette Bromhead, Barry R. Palmer

**Affiliations:** 1 School of Health Sciences, Massey University, Wellington 6021, New Zealand; 2 School of Natural Sciences, Massey University, Palmerston North 4410, New Zealand; 3 School of Veterinary Science, Massey University, Palmerston North 4410, New Zealand; 4 Scion, Rotorua 3010, New Zealand; 5 School of Biological and Chemical Sciences and Ryan Institute, University of Galway, H91 TK33 Galway, Ireland; 6 Waikato Regional Council, Hamilton 3240, New Zealand

**Keywords:** trace elements, heavy metal resistance, antibiotic resistance, bacteria, soil, co-selection, PICT, TRFLP

## Abstract

Accumulation of heavy metals (HMs) in agricultural soil following the application of superphosphate fertilisers seems to induce resistance of soil bacteria to HMs and appears to co-select for resistance to antibiotics (Ab). This study aimed to investigate the selection of co-resistance of soil bacteria to HMs and Ab in uncontaminated soil incubated for 6 weeks at 25 °C in laboratory microcosms spiked with ranges of concentrations of cadmium (Cd), zinc (Zn) and mercury (Hg). Co-selection of HM and Ab resistance was assessed using plate culture on media with a range of HM and Ab concentrations, and pollution-induced community tolerance (PICT) assays. Bacterial diversity was profiled via terminal restriction fragment length polymorphism (TRFLP) assay and 16S rDNA sequencing of genomic DNA isolated from selected microcosms. Based on sequence data, the microbial communities exposed to HMs were found to differ significantly compared to control microcosms with no added HM across a range of taxonomic levels.

## 1. Introduction

Many heavy metals (HMs) have fundamental roles in the metabolism of microorganisms and some act as essential elements in the environment. HMs reach the environment from a variety of different human-related activities (e.g., agricultural, industrial or sewage waste streams) or natural processes (e.g., soil parent materials, volcanic depositions). Accumulation of HMs in soil can lead to bacterial resistance to HMs (HMR) and also co-selection for antibiotic resistance (AbR) [1,2,3]. HMs are generally nondegradable in soil [4], but physicochemical conditions in the soil can strongly influence their mobility and bioavailability [5]. Soil microbiological factors (e.g., bacterial community structure) are also affected by HMs [6]. Soil microcosms are useful tools, on a small and controlled scale, to explore the factors involved in inducing resistance to HMs in soil bacteria [7]. AbR, particularly when it occurs in human and animal pathogens, is a growing threat to health systems around the world [8]. The influence of environmental factors on the development of AbR is often underestimated compared to other factors involved in the spread of AbR such as poor antibiotic stewardship [9].

We previously reported evidence that contamination with Cd and Zn in agricultural soils from New Zealand, due to the addition of superphosphate fertiliser and Zn-containing remedies for the animal disease facial eczema, was associated with selection for HMR and AbR in soil bacteria [3]. In the current study, we aimed to investigate these associations on a more empirical level. Bacterial HMR and AbR profiles and community dynamics were studied in microcosms, containing soil sourced from an uncontaminated site (forest) to which a range of concentrations of Cd, Zn or Hg were added and incubated for 6 weeks. During this incubation, soil samples were monitored for HMR and AbR at regular intervals to investigate whether specific concentrations of HMs, while present in the bacterial biosphere, would induce HMR and/or AbR in bacterial isolates, and could potentially be associated with changes in microbial community structure.

## 2. Results

### 2.1. Heavy Metal Content of Soil and Microcosm Characterisation

Physicochemical characteristics of the sandy loam soil classified as an Immature Orthic Pumice soil used as the base material for the microcosms employed in this study (Table 1), indicated optimal pH and adequate C, N and Olsen P for the current forestry land use of this soil (SINDI soil quality indicator database, Manaaki Whenua-Landcare). The HMs concentrations in the soil from site EW-13 indicated that the soil was uncontaminated.

After dosing the microcosm soil with HMs, the concentration of HMs in drainage leachate samples collected from microcosm trays after 6 h of incubation was analysed by atomic absorption spectrophotometry (AAS). This showed Cd, Zn and Hg were adsorbed strongly to the background soil particles in the microcosms after 6 h of incubation with <1% of the added HMs detected in the microcosms’ drainage (Supplementary Table S1).

### 2.2. HM Resistance in Microcosms

**Figure 1:**
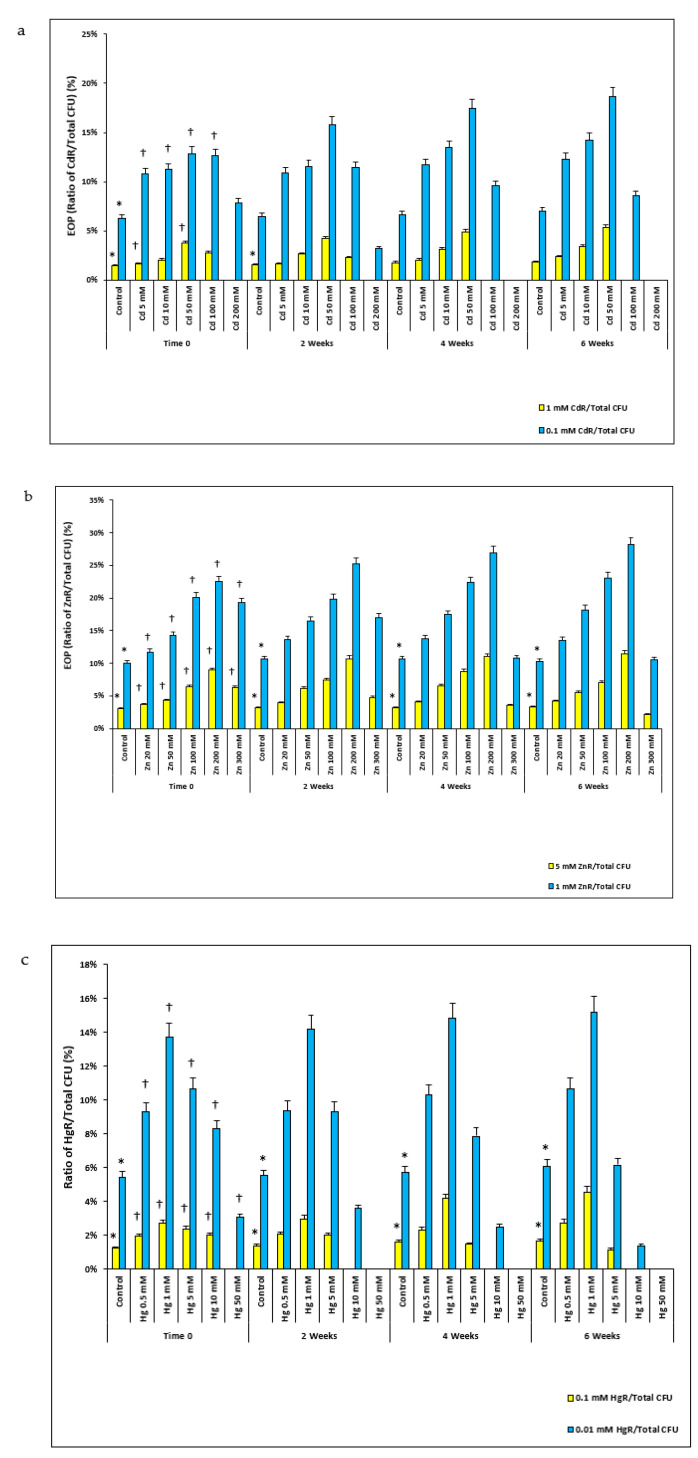
Mean ratios of HMR EOP plotted at 2-week intervals, selected on plates with two concentrations of HM. (**a**) Mean ratios of CdR EOP. (**b**) Mean ratios of Zn resistant EOP. (**c**) Mean ratios of Hg resistant EOP. * *p* < 0.05 compared to the HMR/total bacterial CFU ratios in HM-spiked microcosms; † *p* < 0.05 compared to the HMR/total bacterial CFU ratios in the same HM-spiked microcosms at the 6-week interval.

At time zero, the Cd resistant (CdR) efficiency of plating (EOP) (EOP is the ratio of HMR colony-forming unit (CFU) on HM supplemented plates/total CFU on unsupplemented R2A plates) of soil bacteria from the microcosms was low in the control microcosms and increased with Cd concentration to peak in the Cd 50 mM-spiked microcosm and then decreased in Cd 100 and 200 mM spiked microcosms. CdR EOPs were significantly smaller for control microcosms compared to those from Cd-spiked microcosms (*p* < 0.05). The CdR EOPs in Cd-spiked microcosms at time zero were significantly less compared to those from the same microcosms after 6 weeks. For example, this EOP was about 1.5 times greater for bacteria from the Cd 50 mM spiked microcosms at 6 weeks compared to those at time zero. Similar trends were observed for the Zn-spiked and Hg-spiked microcosms (Figure 1).

### 2.3. Antibiotic Resistance in Microcosms

**Figure 2:**
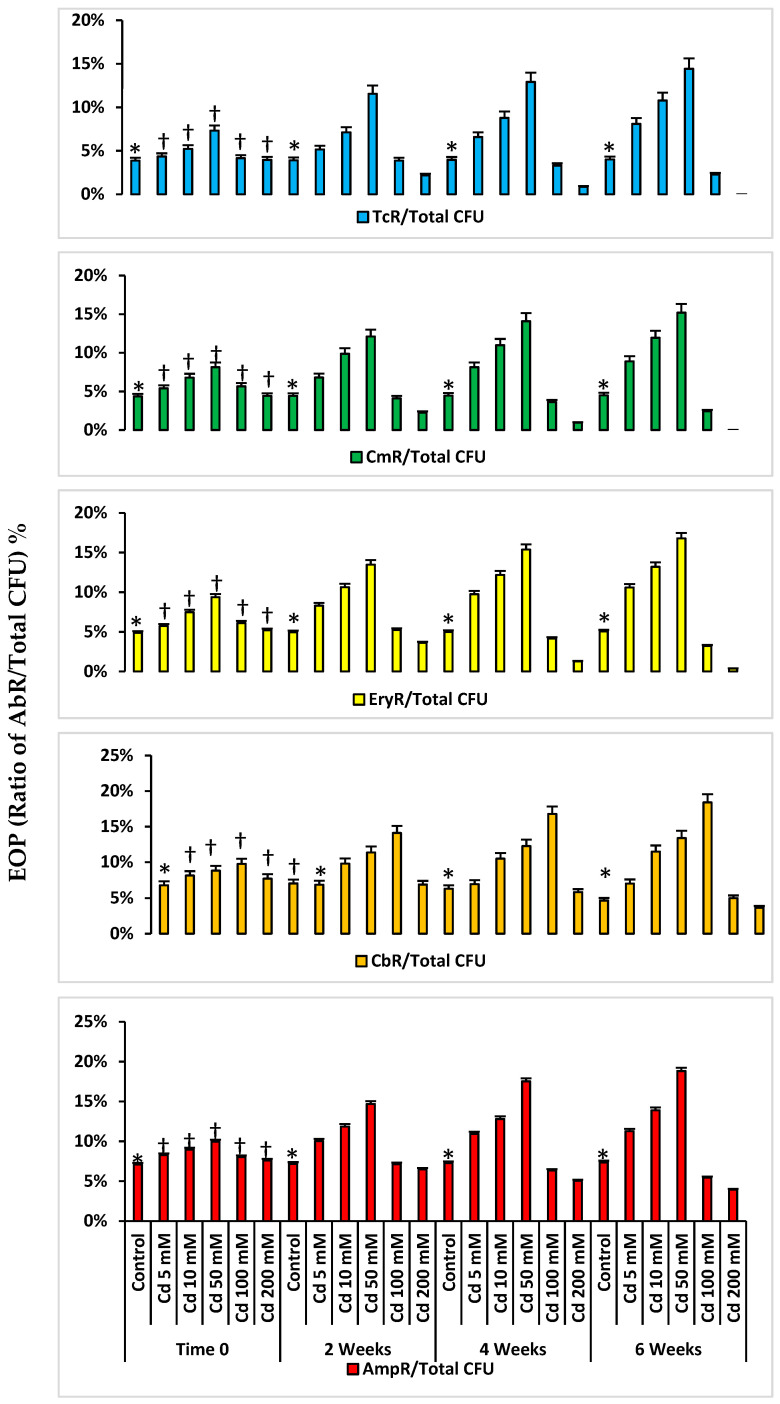
Mean AbR EOPs selected on Abs for Cd-spiked microcosms. * *p* < 0.05 compared to the AbR EOPs in Cd-spiked microcosms; † *p* < 0.05 compared to the AbR EOPs in the same Cd-spiked microcosms at 6 weeks.

The AbR EOPs for the Cd-spiked microcosms at time zero increased from the control to Cd 50 mM spiked microcosms and then decreased in the Cd 100 and 200 mM spiked microcosms (Figure 2). There were significantly lower AbR EOPs for control microcosms compared to those from Cd-spiked microcosms (*p* < 0.05). The AbR EOPs in Cd-spiked microcosms at time zero were significantly less than those from the same microcosms after 6 weeks. For example, the EOP for tetracycline-resistant (TcR) bacteria from the Cd 50 mM spiked microcosm after 6 weeks of incubation was two times greater than that from the same microcosm at time zero (Figure 2). The same trend was observed for the microcosms spiked with a range of Zn and Hg concentrations. The highest EOPs for the other Abs (chloramphenicol: Cm; erythromycin: Ery; carbenicillin: Cb; ampicillin: Amp) were recorded from the same HM-spiked microcosms, which were Cd 50 mM, Zn 200 mM and Hg 1 mM, respectively (Supplementary Figures S1 and S2).

### 2.4. Pollution-Induced Community Tolerance (PICT) Analysis of Microcosms

**Figure 3:**
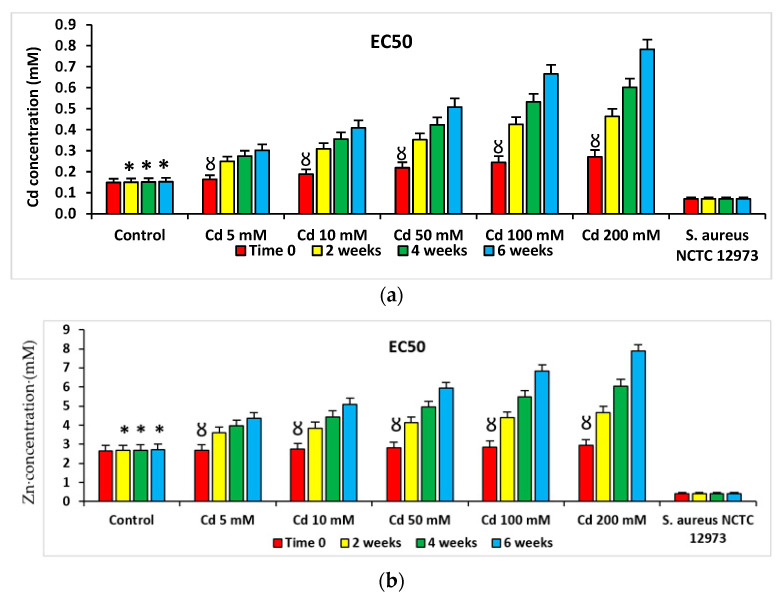

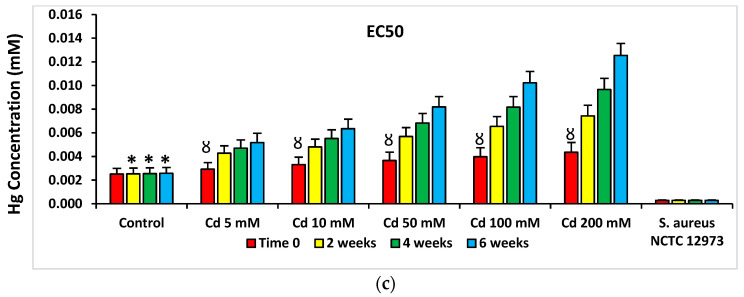
Mean EC50 values of PICT analysis with (**a**) Cd, (**b**) Zn and (**c**) Hg for bacteria from Cd-spiked microcosms. * *p* < 0.05 compared to Cd, Zn or Hg EC50 values for bacteria from Cd-spiked microcosms at the same time point; Ȣ *p* < 0.05 compared to Cd, Zn or Hg EC50 values for bacteria from Cd-spiked microcosms at 2, 4 and 6 weeks. *Staphylococcus aureus* NCTC 12973 was a negative control.

PICT analysis of microbial EC50s for Cd, Zn and Hg from Cd-spiked microcosms showed there were larger Cd EC50 values for bacterial consortia from Cd-spiked microcosms compared to those from control microcosms (*p* < 0.05). Lower EC50 values for Cd were determined for bacterial consortia from Cd-spiked microcosms at time zero compared to those for consortia from the same microcosms at the 2-, 4- and 6-week sampling points (Figure 3). Similar trends were observed for EC50 values of Cd, Zn and Hg for bacterial communities from Zn- and Hg-spiked microcosms (Supplementary Figures S3 and S4).

**Figure 4:**
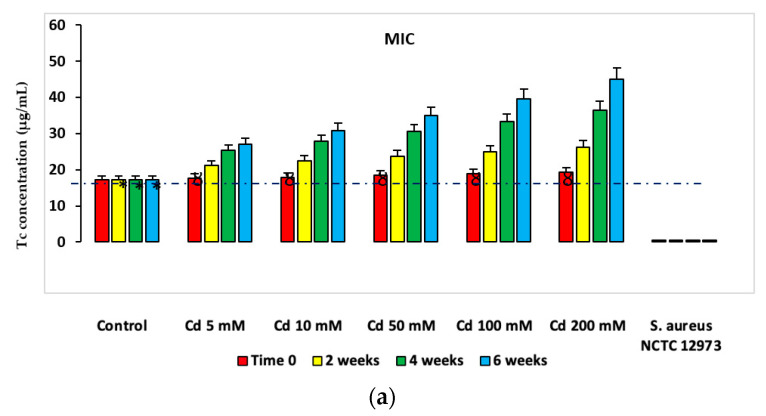

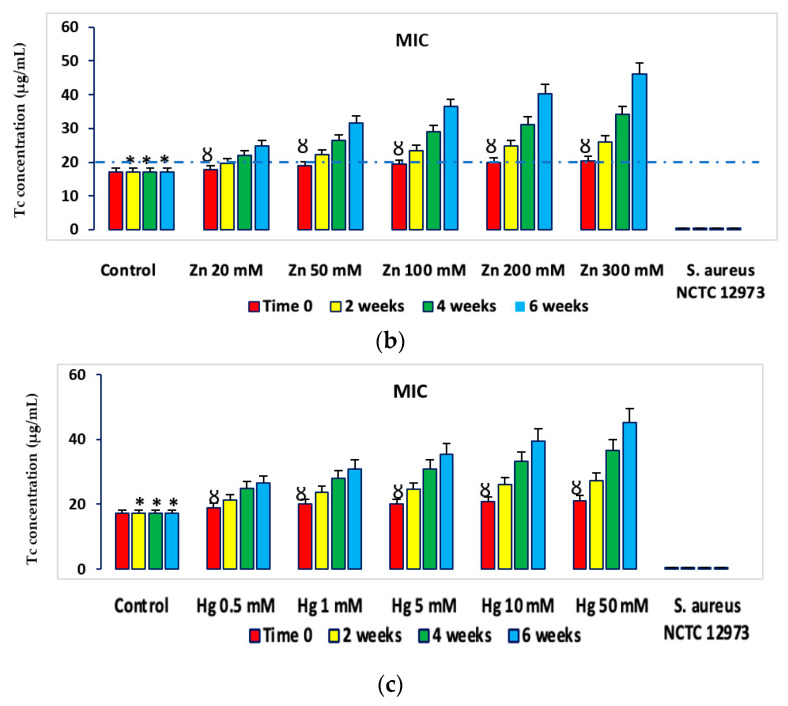
Mean MIC values of PICT analysis with Tc for bacteria from HM-spiked microcosms (**a**) Cd-spiked, (**b**) Zn-spiked and (**c**) Hg-spiked. * *p* < 0.05 compared to Tc MIC for bacteria from Cd-spiked microcosms at the same time point; Ȣ *p* < 0.05 compared to Tc MIC and EC50 values for bacteria from Cd-spiked microcosms at 2, 4 and 6 weeks. The dashed line defines the AbR threshold for soil bacteria. Ab-sensitive *S. aureus* NCTC 12973 was a negative control.

PICT analysis of minimum inhibitory concentrations (MIC) for Tc, Cm, Ery, Cb and Amp for microbial consortia isolated from Cd-spiked microcosms showed there were higher MIC values for Abs from bacterial consortia from Cd-spiked microcosms compared to those from the control microcosms (Figure 4). Figure 4 shows MIC values for Tc for microbial consortia from HM-spiked microcosms and the results for other Abs are shown in the supplementary data (Supplementary Figures S5–S16). Similar trends were determined for MIC values of Ap, Cm, Ery and Cb and for bacterial consortia from Zn- and Hg-spiked microcosms (Supplementary Figures S5–S16). The MIC values determined for microbial consortia (according to EUCAST ECOFF recommendations [10]) from microcosm soil were higher than the 20 µg mL^−1^ threshold defined for Ab resistance in soil bacteria for all of the five Abs at the 2-, 4- and 6-week time points [11,12], while these were lower than this threshold for Tc and Cm at time zero.

### 2.5. Terminal Restriction Fragment Length Polymorphism (TRFLP) Analysis of Soil DNA from Microcosms

#### 2.5.1. Cd-Spiked Microcosms

**Figure 5:**
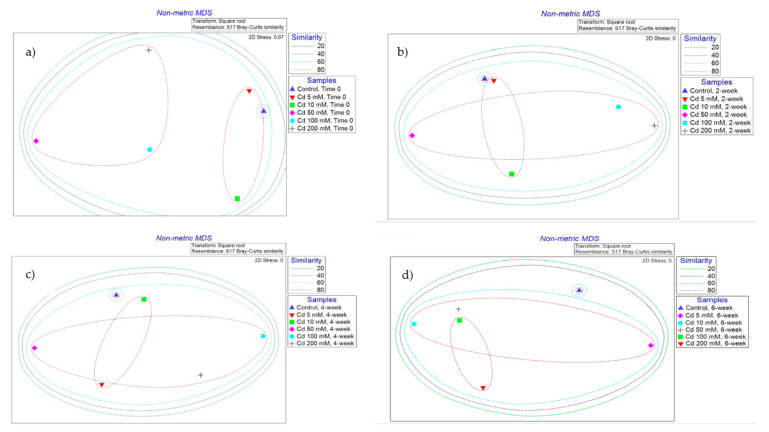
NMDS analysis plot of TRFLP relative peak height for Cd-spiked microcosms bacterial communities’ data, using the Bray–Curtis similarity indices. (**a**) Time zero, (**b**) 2-week incubation at 25 C (**c**) 4-week incubation at 25 C (**d**) 6-week incubation at 25 C. Significant difference (*p* < 0.05) between the clusters specified with >60% of similarity compared to the control microcosms.

Non-metric multidimensional scaling (NMDS) analysis of TRFLP data from Cd-spiked microcosm soil samples showed at time zero there was 60–80% similarity between the bacterial communities from control, 5 and 10 mM Cd-spiked microcosms. In addition, bacterial communities from Cd 50, 100 and 200 mM microcosms were >80% similar at time zero. At week 2, the Cd-spiked communities appear to be becoming more similar. After 4 and 6 weeks, the bacterial communities from most of the Cd-spiked communities were >80% similar and only 60% similar to the control microcosm’s bacterial communities. Three-way ANOVA showed that there were significant differences between the relative abundance of bacterial T-RFs in the control microcosm compared to those from 50, 100 and 200 mM Cd-spiked microcosms’ bacterial communities at all time points (*p* < 0.05) (Figure 5).

#### 2.5.2. Zn-Spiked Microcosms

The NMDS analysis of TRFLP data from Zn-spiked microcosm soil samples revealed that bacterial communities from the control, 20 and 50 mM Zn microcosms were 60–80% similar at time zero and 2 weeks. The bacterial communities from 100, 200 and 300 mM Zn microcosms were >80% similar at time zero and the 2-week time point. After 4 or 6 weeks, the bacterial communities from 20 and 50 mM Zn and 100, 200 and 300 mM Zn microcosms became more similar to each other and less similar to the control. Three-way ANOVA revealed that there were significant differences between the relative abundance of bacterial T-RFs in the control microcosms compared to those from 100, 200 and 300 mM Zn at time zero and the 2-week time point and also all of the Zn-spiked microcosm at time 0 and after 2, 4 and 6 weeks of incubation (*p* < 0.05) (Figure S17).

#### 2.5.3. Hg-Spiked Microcosms

The NMDS analysis of Hg-spiked microcosm soil samples’ TRFLP data revealed that there was >80% similarity between bacterial communities from control, 0.5 and 1 mM Hg microcosms, and also between communities from 5, 10 and 50 mM Hg microcosms at time zero. The similarity of the communities from control microcosms to other Hg-spiked microcosms changed to >60% after 2 weeks. The bacterial communities from 0.5, 1, 5 and 10 mM Hg microcosms showed >80% similarity after 2 weeks. In addition, the communities from 1, 5, 10 and 50 mM Hg were >80% similar at the 2-week time point. After 6 weeks, the similarity of the control microcosm’s communities to other microcosms was reduced by >40%. The communities from 0.5 and 1 mM Hg microcosms were >80% similar after 4 and 6 weeks. Moreover, communities from 5, 10 and 50 mM Hg microcosms showed >80% similarity. Three-way ANOVA showed there were significant differences between the relative abundance of bacterial T-RFs in the control microcosms compared to those from Hg-spiked microcosms at the 2-, 4- and 6-week time points (*p* < 0.05) (Figure S18).

### 2.6. 16S rRNA Gene Analysis of Microbial Community Structure

**Figure 6:**
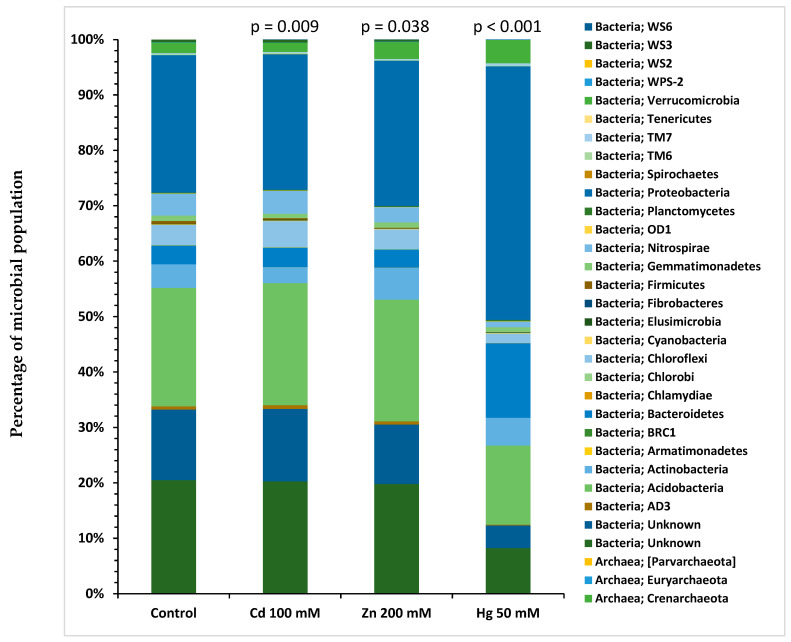
Assignment of 16S rRNA gene sequences to microbial phyla for selected HM-spiked microcosms (Cd 100 mM, Zn 200 mM and Hg 50 mM, control) soil samples after 6 weeks of incubation. *p*-values represent differences between data from HM and control samples.

Analysis of the metagenome by NGS of 16S rDNA genes from selected microcosm soil samples was performed using QIIME 1 software [13]. Soil samples analysed were from control, Cd 100 mM, Zn 200 mM and Hg 50 mM spiked microcosms taken after 6 weeks of incubation. These microcosms were analysed because they represented extremes of selection across the ranges of HM concentrations used, without very high toxicity for Cd and Zn. There were significant differences between the number of taxonomically classified reads related to each bacterial phylum for the HM-spiked soil samples compared to those for the control sample (*p* < 0.05). Figure 6 illustrates the diversity of the microbial communities in the selected microcosm soils at the level of the phylum. The most abundant phyla determined in the microcosm soil samples were *Proteobacteria*, *Bacteroidetes*, *Actinobacteria*, *Acidobacteria* and *Chloroflexi*. Table 2 shows a comparison of the number of 16S rDNA gene reads in Cd-, Zn- and Hg-spiked microcosm soil samples for the different taxonomic levels, including phylum, class, order, family, genus and species. The proportion of *Acidobacteria* in the Hg-spiked microcosm was reduced compared to the control microcosm.

## 3. Discussion

Plate culturing of bacteria isolated from microcosms revealed that EOPs for HMR in control microcosms were significantly lower than those from HM-spiked microcosms. Chu (2018) [14] described the presence of HMs in soil as the most extreme factor by which bacterial populations and communities are changed compared to other types of contamination. There are reports indicating that the presence of high levels of HMs in soil for a short period of time can induce resistance in the bacterial communities, although adaptation to HMs stresses can also happen over longer periods of time, especially with levels much higher than bacterial tolerance thresholds as used in the microcosms described here [14]. Caliz et al. (2011) [15] reported that the bioavailability of HMs is a substantial factor in effectively inducing bacterial resistance, along with the potential toxicity of the HMs. High levels of exchangeable HMs can lead to higher levels of bacterial resistance to these HMs, as well as other HMs, and a range of antibiotics owing to common resistance mechanisms (e.g., common cellular efflux pumps and presence of resistance genes on the same genetic elements) [16]. Low pH can also increase the bioavailability of HMs, but this is unlikely to have been a factor in the soil used as a starting material in the trials presented here.

After HM spiking of the microcosm soils, the analysis of HMs concentrations in the microcosms’ leachate after 6 h of incubation showed strong adsorption of Cd, Zn and Hg ions to the soil particles. This demonstrated that HM levels in the microcosms’ soils were raised significantly by spiking and only a small amount of each HM’s ions were detected in the microcosms’ leachate. The levels of organic matter in soil and its pH are likely to be the main factors involved in HMs’ adsorption to the soil particles and may affect the levels of bioavailability of HMs in soils [17].

The levels of Ab resistance in bacteria isolated from microcosms were significantly higher in the HM-spiked microcosms’ soil compared to those from control microcosms. This suggests that inducing different levels of HM resistance in bacterial consortia in the presence of these HMs in soil can lead to co-resistance to various Abs [16,18,19,20,21]. This data suggests that the concentrations of Cd and/or Zn contamination found in some agricultural soils (range: Cd: 0.00–2.52 mg/kg; Zn: 10–250 mg/kg) in New Zealand are high enough to select for AbR in soil bacteria, as determined by our results in the current study and our previous report [3]. While the current study did not include the addition of phosphate fertiliser to test microcosms, the data generated from microcosms spiked with Cd, a major contaminant in the superphosphate fertiliser used in New Zealand, are suggestive that similar selection for HMR and AbR bacteria could result from soil fertilised with phosphate fertilisers. Given the size of the areas subjected to phosphate fertiliser amendment for agricultural purposes in New Zealand and worldwide, the potential for enrichment of resistant bacteria and antibiotic resistance genes is large and may represent an under-appreciated threat to human and animal health. It is suggested that bacterial resistance to Cd and Zn would also result in greater levels of resistance to Hg due to common mechanisms (e.g., cellular efflux pumps) [22]. The levels of HMR EOPs in microcosms increased from time zero to the 6-week time point.

In the context of New Zealand’s agricultural soils, two points are worth noting. First, soil contamination by Hg above recommended guidelines is rare and generally confined to specific urban commercial/industrial sites, some intensively cropped horticulture sites (likely due to historical pesticide use) and some geothermally influenced areas. Second, fluorine (F) [17] and uranium (U) [23] are two other significant trace element contaminants which have been accumulating in New Zealand agricultural soils through long-term application of phosphate fertilisers, in addition to Cd and the phosphorus (P) itself. In a real-world agricultural soil setting, the picture is one of increased fertility coupled with microbial co-exposure to gradually increasing concentrations of available Cd, F and U (from phosphate fertilisers) and Zn (primarily from facial eczema remedies), and land-use factors that alter physiochemical conditions such as increased soil compaction under livestock farming. Other authors have explored the impact of land-use history on changes in soil microbial communities [24,25].

The results of PICT analysis of bacterial consortia for samples from microcosm soils revealed that their EC50 values were greater for HMs and Abs’ for HM-spiked microcosms compared to those from the control microcosm soil. It is interpreted that added levels of HMs in microcosms soils led to higher levels of HM resistance in the microcosms’ soil microbiota. These results and previous reports [24,25] suggest that similar processes occur in the wider environment [26].

Change in the structure of bacterial communities was assessed using the techniques of TRFLP analysis and 16S rDNA sequencing of total DNA isolated from soil samples from the microcosms. Resources precluded sequence analysis of DNA except from samples taken after 6 weeks of incubation from selected microcosms (i.e., those thought to represent where the greatest selection for HMR and AbR had taken place). However, both techniques demonstrated that significant changes in community structure had taken place in the presence of elevated levels of HMs. Changes were particularly marked in the presence of Hg, which may be due to the greater relative toxicity of Hg compared to Cd or Zn. In a similar experimental design, Fu et al. (2023) [27] found a marked reduction in the proportion of Acidobacteria, as we did, at high HM levels.

In conclusion, this study showed that the EOPs for HMR and AbR were higher in soil samples with mid-range levels of HMs compared to those with lower concentrations and the highest concentrations used. The bacteria subjected to the selective pressure of HMs in soil showed higher resistance to both HMs and Abs. These findings support the hypothesis that levels of HM resistance respond to direct selection pressure in the presence of elevated levels of HMs in the environment, while Ab resistance in soil bacteria is increased via indirect selection, probably by co-resistant and cross-resistant mechanisms.

## 4. Materials and Methods

### 4.1. Physicochemical Properties of the Soil Sampling Sites

Soil samples collected from a site near Taupō with a long history of plantation forestry and a sandy loam soil structure, classified as an Immature Orthic Pumice soil in the New Zealand Soil Classification [28] and as a Typic Udivitrand in Soil Taxonomy [29], were used as the base soil for the microcosms. Chemical analysis of representative samples indicated the concentration of the three HMs of interest in the soil used as starting material for the microcosms.

### 4.2. Microcosms’ Leachate HM Concentration Analysis

Metal concentrations of drainage fluids collected from microcosm trays were analysed using atomic absorption spectrophotometry (AAS). For both flame AAS (FAAS) and graphite-furnace (GFAAS) determinations, the instrument used was an Analytik Jena ContrAA 700 High-Resolution Continuum Source Atomic Absorption Spectrometer for Flame and Graphite Furnace. Based on the initial metal concentrations added to the microcosms, two different methods of metal concentration analysis were performed. FAAS (air/acetylene) was used for higher-level Cd and Zn determinations [30], and GFAAS was used when quantification was required below the FAAS detection limit (*ca.* 0.1 mg/L for both Cd and Zn), and for Hg (for which FAAS does not apply). The detection limits by GFAAS were Cd: 0.0003; Zn: 0.0001; Hg: 2.5 mg/L [31].

After collection and prior to analysis, samples were preserved by adding sufficient analytical reagent (AR) nitric acid (Sigma-Aldrich, Auckland, New Zealand) to achieve a final acid concentration of 0.5% *v*/*v*. The analysis was carried out against suitably prepared matrix-matched standards on a high-resolution Continuum Source AAS. Testing carried out in graphite-furnace mode made use of appropriate matrix modifiers. Determinations were carried out at wavelengths of 228.8018 nm for Cd, 213.8570 nm for Zn and 253.6519 nm for Hg, which are primary absorption lines for the three elements [32]. Standard solutions were prepared by dilution from ICP Multi-Element Standard Solution IV for Cd and Zn, and 0.1 *M* HgCl_2_ (Sigma-Aldrich, Auckland, New Zealand) solution for Hg. Standards and blank solutions were prepared using distilled water and preserved with AR nitric acid (final strength 0.5% *v*/*v*).

### 4.3. Microcosms

Polypropylene boxes with 500 g capacity (dimensions: L = 20, W = 15, H = 5 cm) were used as containers for the microcosms with 10 × 1 mm diameter drainage holes in the bottom, and 5 × 10 mm ventilation holes in the lids to avoid excess soil moisture. Soil samples (300 g) were added to each microcosm container after the removal of large stones, debris etc. by sieving (aperture = 5 mm). Each microcosm had a tray to collect the drainage fluid.

Microcosms were incubated for a total period of 6 weeks at 25 °C. Topsoil from a site near Taupō, with no or limited HM pollution history, was used as a starting material. The site is included in the Waikato Regional Council’s Regional Soil Quality Monitoring Programme [17]. The soil was collected from the upper soil horizon at 0–10 cm depth during June 2015. Each prepared microcosm was spiked by one of the five concentrations of CdCl_2_, ZnSO_4_·7H_2_O or HgCl_2_ in triplicate to reach the final concentrations of Cd ions of 5, 10, 50, 100 and 200 mM (~560–22,500 ppm); Zn ions: 20, 50, 100, 200 and 300 mM (1300–19,600 ppm); or Hg ions: 0.5, 1, 5, 10 and 50 mM (100–10,000 ppm). Three microcosms were assigned as controls and received only sterile distilled water with no metal added. HM stocks were prepared as previously described [3]. Aliquots of 300 mL of sterile distilled water were spiked with the appropriate amount of HM stock solution and this was sprayed onto the microcosms’ soil. Microcosms were left for 6 h and then their drainage fluids were collected for metal concentration analysis by AAS.

The soil of the microcosms was mixed every week using sterile spatulas [33]. Weekly moisture measurement of the microcosms’ soils was performed and adequate water was added to maintain constant soil moisture of 20 ± 5% (the original soil sample’s moisture content [34]). Duplicate 10 g of soil samples were taken after 6 weeks of incubation, one for PICT testing and the other for DNA extraction for TRFLP assay and stored at 4 °C and −20 °C, respectively.

### 4.4. Plate Culture

To sample heterotrophic aerobic plate counts of bacteria in the soil of microcosms, 10 g (dry weight) of soil from each microcosm was mixed with 90 mL of sterile PBS buffer and agitated in a shaking incubator at 200 rpm for 1 h. Aliquots of 100 µL of 10-fold serial dilutions of this suspension were cultured on R2A plates containing one of two concentrations of HMs (0.1 and 1 µg mL^−1^ for Cd and Zn, and 0.01 and 0.1 µg mL^−1^ for Hg) or one concentration of Abs (20 µg mL^−1^ for Tc and Cm, 100 µg mL^−1^ for Cb and Ery, and 200 µg mL^−1^ for Amp, all Abs from Sigma-Aldrich Auckland) as well as control plates (no added metal or Ab) (chosen to represent a range of antibiotic classes) in triplicate at time zero and two-weekly intervals during incubation at 25 °C [15,33,34]. Total CFUs were calculated for each soil sample and efficiency of plating (EOP) percentages were calculated, the ratio of CFU count on media with added HM or Ab to CFU on unamended medium. pH measurement of soils was performed at time zero and every other sample round and compared to control microcosms [15,35,36].

### 4.5. Pollution-Induced Community Tolerance (PICT) Analysis

The tolerance of a community of microbes to a range of antimicrobial agents at various concentrations was determined by a microtitre plate culturing-dependent method called pollution-induced community tolerance (PICT) [37,38]. The PICT test was carried out for soil samples taken from the microcosms. R2A broth was used as a common substrate for soil bacterial communities. To achieve an inoculum at the desired density, optical density (OD) was measured spectrophotometrically using a McFarland 0.5 turbidity standard [39]. To reach the desired number of cells, in liquid culture of 5 × 10^5^ mL^−1^ suitable dilutions in R2A medium were performed. A 100 µL volume of PBS with ~5 × 10^5^ bacterial cells per mL was added to each well of a microtiter plate containing 99 µL of 2 × R2A broth [40]. A 1 µL aliquot of HMs or Abs was added to the allocated wells in triplicate, as were negative and positive controls.

Ab-sensitive *S. aureus* NCTC 12973 was added to the experiment batches as quality control, and the resulting data were compared with the EUCAST databases [18,35]. Incubation was performed in a shaking incubator at 25 °C and 200 rpm for 72 h. OD readings from each well were recorded at time zero, before inoculation of wells with antimicrobials to monitor the possible OD changes due to antimicrobial agents addition, and at 6 h intervals [41]. EC50s were calculated for each batch of results. A half-filled 5 L beaker of water was put in the shaking incubator to provide enough humidity to prevent the liquid culture from drying out.

### 4.6. Soil DNA Extraction

Total genomic DNA of soil samples was extracted to allow 16S rDNA sequencing and terminal restriction fragment length polymorphism (TRFLP) analysis. DNA was extracted from soil samples using Mo Bio PowerSoil^®^ DNA Isolation Kits following the manufacturer’s protocol. Quantitative and quality assays control of the extracted DNA samples were achieved by 1.5% agarose gel electrophoresis in 0.5% TBE buffer, and spectrophotometry for A260/280 nm and A260/230 nm ratios to detect impurities [42].

### 4.7. Terminal Restriction Fragment Length Polymorphism (TRFLP)

Quality control of DNA samples was performed by trial PCR reactions using unlabelled 16S rDNA gene-specific primers with the same sequence as the labelled ones used for sequencing (Thermo Fisher Scientific), and 2% agarose gel electrophoresis (100 V, 40 min). A negative control containing no DNA template was included with these PCR reactions and with reactions using labelled primers. The 63F forward primer was labelled at its 5′-end with 6-FAM™ phosphoramidite dye-labelled (blue) —5′-CAG GCC TAA CAC ATG CAA GTC-3′— [42] and the 1087R reverse primer was VIC^®^ phosphoramidite dye-labelled (green) —5′-CTC GTT GCG GGA CTT AAC CC-3′— [43]. PCR product quality was assessed by ethidium bromide staining after running the PCR product on a 2% agarose gel (100 V, 40 min) [44].

A 25 µL aliquot of PCR product (~200 ng PCR product) was digested with *Msp*I (Thermo Fisher Scientific) at 37 °C for 3 h and subsequent 30 min enzyme deactivation at 65 °C and a final fast cooling at 4 °C [44]. This step generated fluorescently labelled terminal restriction fragments. Restriction fragment lengths were measured by the detection of terminal fluorescent labelled fragments analysed by the ABI3730 Capillary Genetic Analyser at Massey Genome Service, Massey, University, Palmerston North. The output was a series of peaks of various sizes and heights that represents the profile of each sample [45]. The enzyme digestion products were visualised for quality control purposes by analysing on a 2% agarose gel run at 100 V for 40 min. A negative control without a DNA template was included for the PCR reactions.

TRFLP data analysis was performed with GeneMapper^®^ software v.4.1 for peak analysis and PRIMER v.7 (Plymouth Marine Laboratory) for NMDS analysis of the relative abundance of terminal restriction fragments as a proportion of a total peak height of all the terminal restriction fragments (T-RFs) in that profile to provide a comprehensive 1000019290fingerprint from metagenomics samples [45]. Three-way ANOVA was used for statistical analysis to compare the abundance of the T-RFs between samples [44].

### 4.8. 16S rDNA Sequencing

In order to investigate the diversity of the bacterial communities in soil samples, 16S rDNA sequencing was performed. Total genomic DNA samples from microcosms spiked with Cd 100, Zn 200 and Hg 50 µg mL^−1^ were sent to the Massey Genome Service hub of NZ Genomics Ltd., Massey University, Palmerston North. Illumina 16S V3-V4 rRNA library preparation and the Illumina MiSeq 16S rDNA sequencing test were performed on these samples. The quality control for 1S rDNA sequencing and the subsequent analysis was achieved with SolexaQA++ [46] and QIIME 1 [13] software tools to generate a comprehensive taxonomic overview of the soil samples’ microbial communities.

## Figures and Tables

**Table 1:** Soil samples’ physicochemical properties for the soil used in microcosms.

Soil Property	Measurement ^1^
pH	5.90 ± 0.065
Total C (%)	5.10 ± 0.35
Total N (%)	0.36 ± 0.022
C:N	14.2 ± 0.97
Olsen P	40.0 ± 4.72 ^2^
Cd	0.13 ± 0.018 ^2^
Hg	0.05 ± 0.009 ^2^
Zn	19.4 ± 3.20 ^2^
Fe	4500 ± 460 ^2^
P	1090 ± 120 ^2^

^1^ Detection limits: Cd and Hg: 0.01 mg kg^−1^; Zn: 0.4 mg kg^−1^; Fe and P: 40 mg kg^−1^; Olsen P: 1 mg kg^−1^; C: 0.01%; N: 0.009%; ^2^ mg kg^−1^ of dry soil.

**Table 2:** Comparison of microbial 16S rRNA gene reads in various taxonomical levels of Cd-, Zn- and Hg-spiked microcosms compared to the control microcosm soil.

Taxonomy Levels	Cd 100 mM Spiked Microcosms	Zn 200 mM Spiked Microcosms	Hg 50 mM Spiked Microcosms
Phylum	*p* = 0.009	*p* = 0.038	*p* < 0.001
Class	*p* = 0.008	*p* = 0.030	*p* < 0.001
Order	*p* = 0.007	*p* = 0.020	*p* < 0.001
Family	*p* = 0.006	*p* = 0.015	*p* < 0.001
Genus	*p* = 0.006	*p* = 0.010	*p* < 0.001
Species	*p* = 0.006	*p* = 0.010	*p* < 0.001

## Data Availability

Data are available from https://eds.s.ebscohost.com/eds/detail/detail?vid=1&sid=b72ec5a7-a1cb-477e-8568-90cbbf8c7816%40redis&bdata=JkF1dGhUeXBlPXNzbyZzaXRlPWVkcy1saXZlJnNjb3BlPXNpdGU%3d#AN=mul.oai.edge.massey.folio.ebsco.com.fs00001086.145a55db.ebe3.5e5b.b06c.8a7ab61bb345&db=cat09011a, accessed 6 April 2023.

## Supplementary Materials

The following supporting information can be downloaded at: https://www.mdpi.com/article/10.3390/antibiotics12040772/s1, Figure S1: Mean ratios of AbR/total bacterial CFU, selected on Abs (20 µg mL^−1^ of Cm, 100 µg mL^−1^ of Ery, 100 µg mL^−1^ of Cb and 200 µg mL^−1^ of Amp) for Zn-spiked microcosms. Figure S2. Mean ratios of AbR/total bacterial CFU, selected on Abs (20 µg mL^−1^ of Cm, 100 µg mL^−1^ of Ery, 100 µg mL^−1^ of Cb and 200 µg mL^−1^ of Amp) for Hg-spiked micrcosms. Figure S3. Mean MIC and EC50 values of PICT analysis with Tc for bacteria from Zn-spiked microcosms. Figure S4. Mean MIC and EC50 values of PICT analysis with Tc for bacteria from Hg-spiked microcosms. Figure S5. Mean MIC and EC50 values of PICT analysis with Cm for bacteria from Cd-spiked microcosms. Figure S6. Mean MIC and EC50 values of PICT analysis with Ery for bacteria from Cd-spiked microcosms. Figure S7. Mean MIC and EC50 values of PICT analysis with Cb for bacteria from Cd-spiked microcosms. Figure S8. Mean MIC and EC50 values of PICT analysis with Amp for bacteria from Cd-spiked microcosms. Figure S9. Mean MIC and EC50 values of PICT analysis with Cm for bacteria from Zn-spiked microcosms. Figure S10. Mean MIC and EC50 values of PICT analysis with Ery for bacteria from Zn-spiked microcosms. Figure S11. Mean MIC and EC50 values of PICT analysis with Cb for bacteria from Zn-spiked microcosms. Figure S12. Mean MIC and EC50 values of PICT analysis with Amp for bacteria from Zn-spiked microcosms Figure S13. Mean MIC and EC50 values of PICT analysis with Cm for bacteria from Hg-spiked microcosms Figure S14. Mean MIC and EC50 values of PICT analysis with Ery for bacteria from Hg-spiked microcosms. Figure S15. Mean MIC and EC50 values of PICT analysis with Cb for bacteria from Hg-spiked microcosms. Figure S16. Mean MIC and EC50 values of PICT analysis with Amp for bacteria from Hg-spiked microcosms. Figure S17. NMS analysis plot of TRFLP relative peak height for the Zn-spiked microcosm soil’s bacterial communities’ data, using the Bray-Curtis similarity index. Figure S18. NMS analysis plot of TRFLP relative peak height for the Hg-spiked microcosm soil’s bacterial communities’ data, using the Bray-Curtis similarity index. Table S1: Leachate HM concentration analysis of 6WM after 6 h of incubation.
