# Supplementary material for: Co-Selection of Bacterial Metal and Antibiotic Resistance in Soil Laboratory Microcosms
Source: Antibiotics (Basel). 2023 Apr 18;12(4):772. doi: 10.3390/antibiotics12040772 (PMC10135173; doi:10.3390/antibiotics12040772)
Supplement: Supplementary file 1 [file antibiotics-12-00772-s001.zip › antibiotics-2281254-supplementary.pdf]

Supplementary Materials

Table S1. Leachate HM concentration analysis of 6WM after 6 hours of incubation. .

Microcosm	Microcosm HM conc. (mg L^{-1})	Original volume of added HM stock (L)	Total HM originally added (mg)	Total HM left in drainage after adsorption (mg)	Total HM lost by adsorption (mg)	Increase of HM conc. in soil (mg kg^{-1})	HM total adsorption (%)
Cd-spiked	Control	0.3	0	0.0026	-0.0026	0	0
	1	0.3	0.3	0.0087	0.29	1	97.09
	5	0.3	1.5	0.011	1.48	5	99.26
	10	0.3	3	0.019	2.98	10	99.36
	50	0.3	15	0.084	14.91	50	99.43
	100	0.3	30	0.19	29.80	99	99.36
Zn-spiked	Control	0.3	0	0.011	-0.011	0.0	0
	20	0.3	6	0.043	5.95	19.9	99.26
	50	0.3	15	0.057	14.94	49.8	99.61
	100	0.3	30	0.065	29.93	99.8	99.78
	200	0.3	60	0.22	59.77	199.3	99.62
	300	0.3	90	0.40	89.59	298.6	99.54
Hg-spiked	Control	0.3	0	0	0	0.0	0
	0.5	0.3	0.15	0.0038	0.14	0.5	98.93
	1	0.3	0.3	0.0077	0.29	1.0	98.75
	5	0.3	1.5	0.038	1.46	4.9	98.55
	10	0.3	3	0.077	2.92	9.7	98.52
	50	0.3	15	0.38	14.61	48.7	98.50

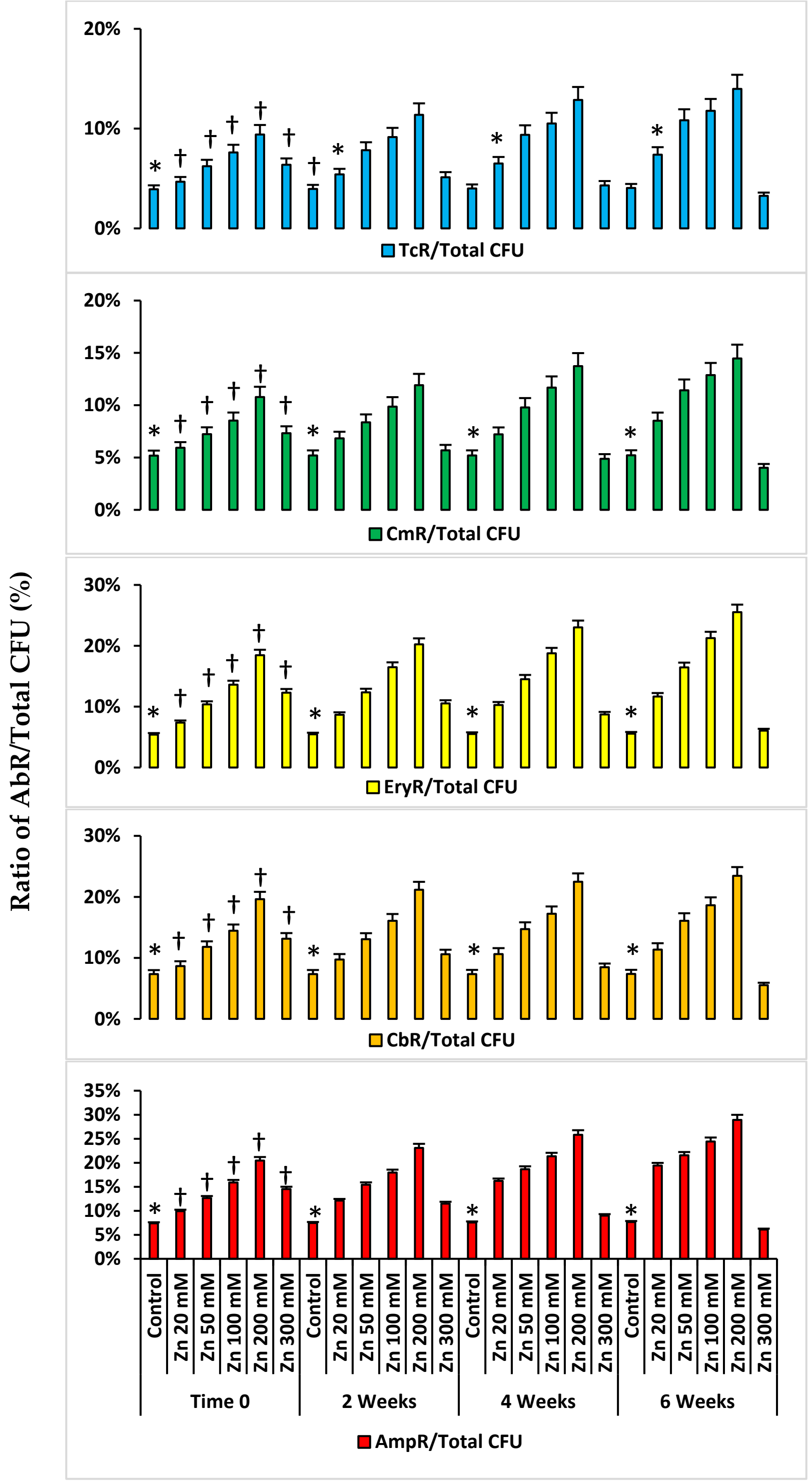

Figure S1. Mean ratios of AbR/total bacterial CFU, selected on Abs (20 μg mL^{-1} of Cm, 100 μg mL^{-1} of Ery, 100 μg mL^{-1} of Cb and 200 μg mL^{-1} of Amp) for Zn-spiked microcosms. *$p<0.05$ compared to the AbR/total bacterial CFU ratios in 6WZnM; †$p<0.05$ compared to the AbR/total bacterial CFU ratios in the same 6WZnM at the 6-week interval.

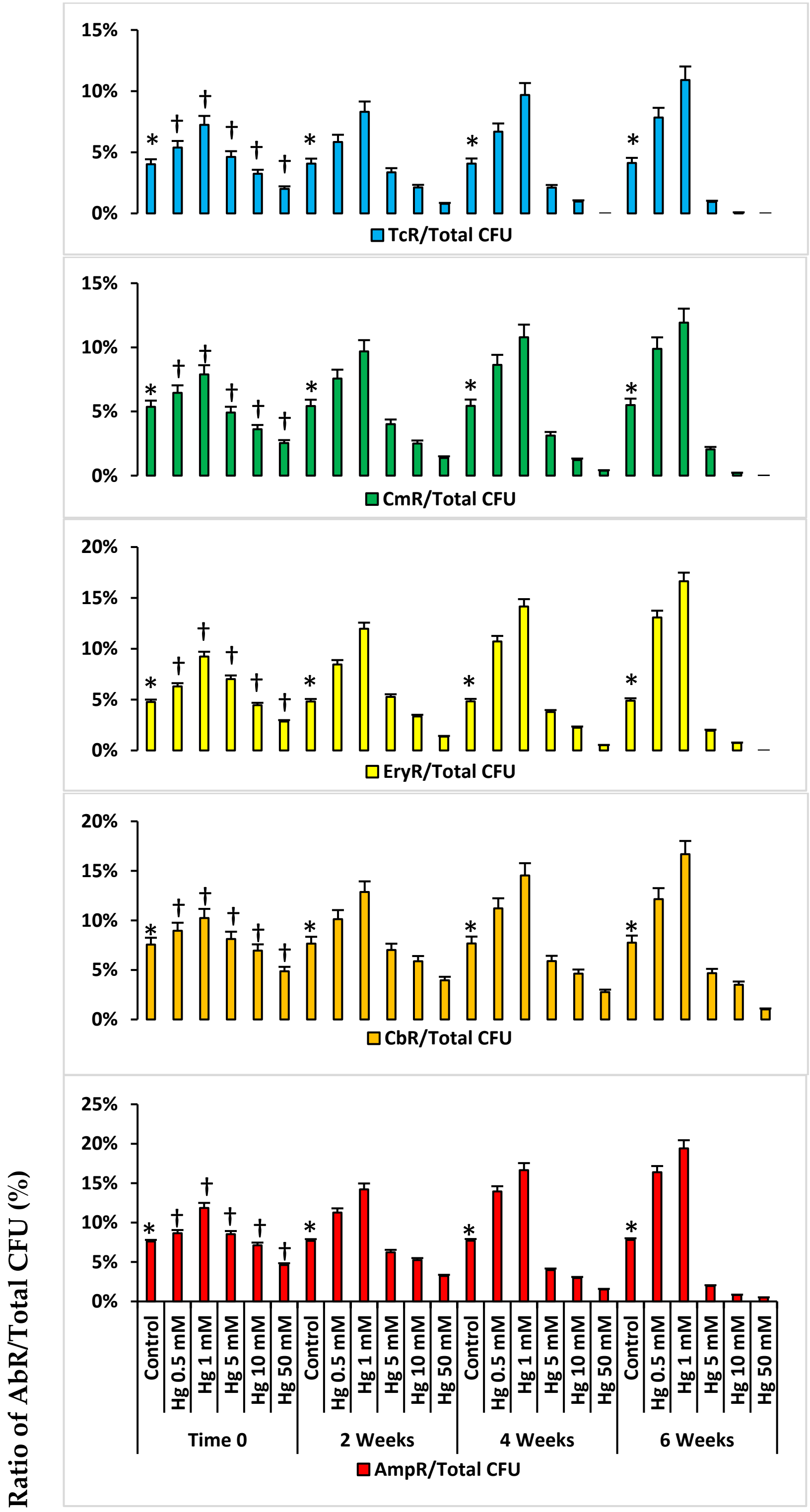

Figure S2. Mean ratios of AbR/total bacterial CFU, selected on Abs (20 μg mL^{-1} of Cm, 100 μg mL^{-1} of Ery, 100 μg mL^{-1} of Cb and 200 μg mL^{-1} of Amp) for Hg-spiked micrcosms. **$p<0.05$* compared to the AbR/total bacterial CFU ratios in 6WHgM; †*$p<0.05$* compared to the AbR/total bacterial CFU ratios in the same 6WHgM at the 6-week interval.

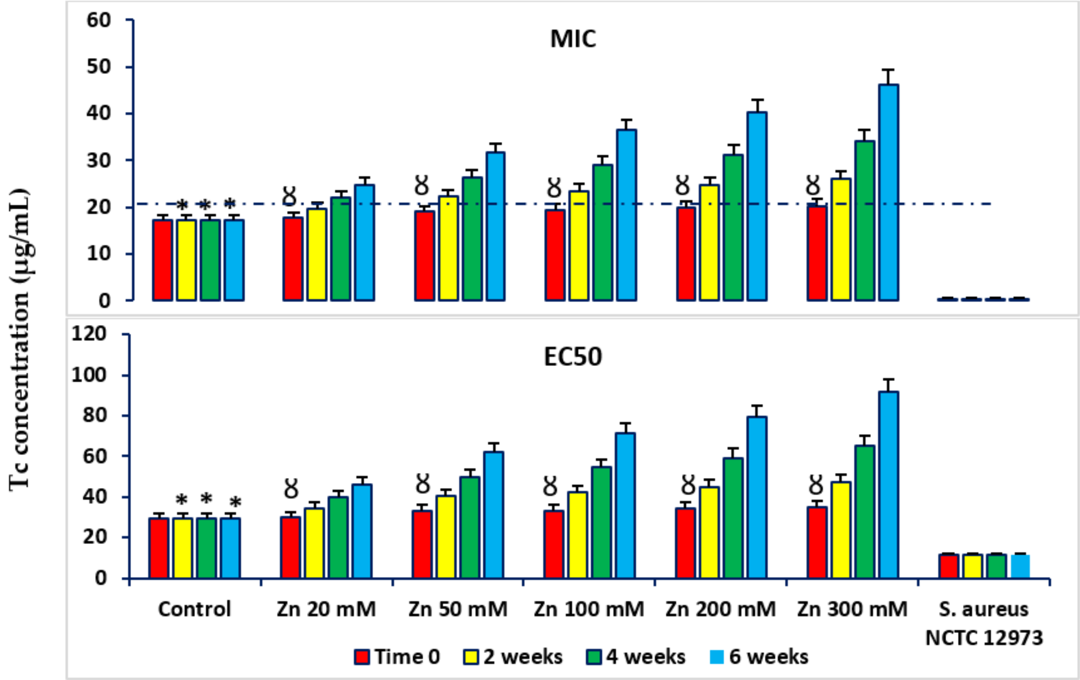

Figure S3. Mean MIC and EC50 values of PICT analysis with Tc for bacteria from Zn-spiked microcosms. *$p < 0.05$ compared to Tc MIC and EC50 values for bacteria from Zn-spiked microcosms at the same timepoint; 8$p < 0.05$ compared to Tc MIC and EC50 values for bacteria from Zn-spiked microcosms at 2, 4 and 6 week. The dash line defines AbR level of soil bacteria.

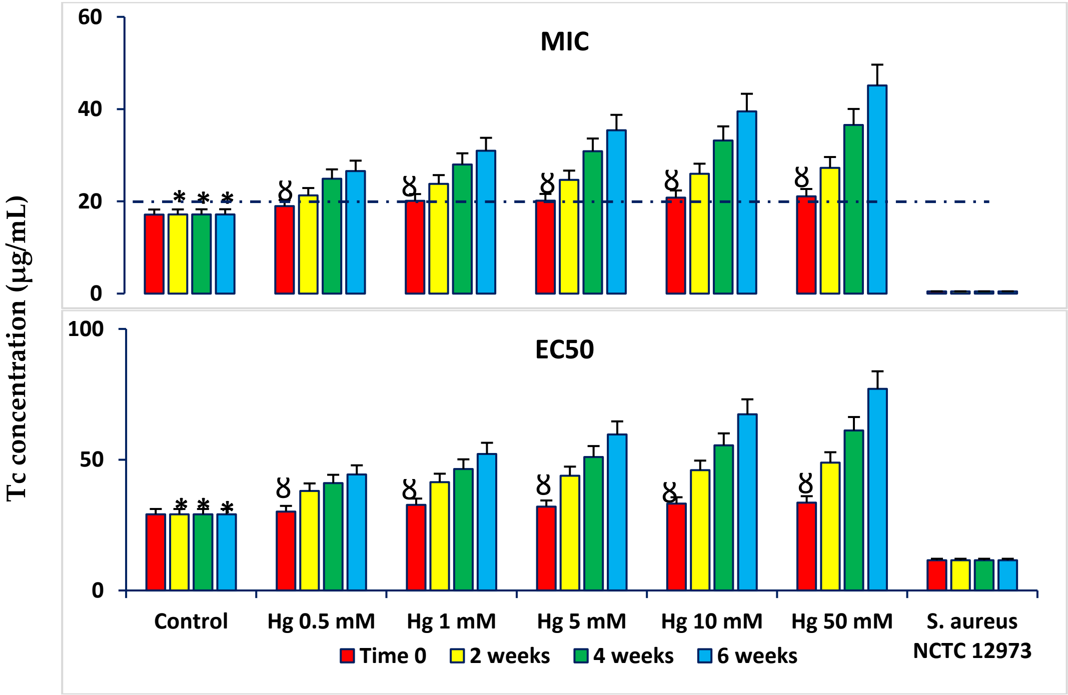

Figure S4. Mean MIC and EC50 values of PICT analysis with Tc for bacteria from Hg-spiked microcosms. *$p < 0.05$ compared to Tc MIC and EC50 values for bacteria from Hg-spiked microcosms at the same timepoint; 8$p < 0.05$ compared to Tc MIC and EC50 values for bacteria from Hg-spiked microcosms at 2, 4 and 6 week. The dash line defines AbR level of soil bacteria.

Analysis Using Abs

As discussed in Section 5.4.1 for bacteria from 6WCdM, there were significantly greater Abs MIC and EC50 values for bacteria from 6WZnM and 6WHgM compared to those from control microcosms ($p < 0.05$). Lower Abs MIC and EC50 values were determined for bacteria from 6WZnM and 6WHgM at Time 0 compared to those for bacteria at 2, 4 and 6 weeks intervals (Figures A3.30-A3.37 and Tables A3.32-A3.43).

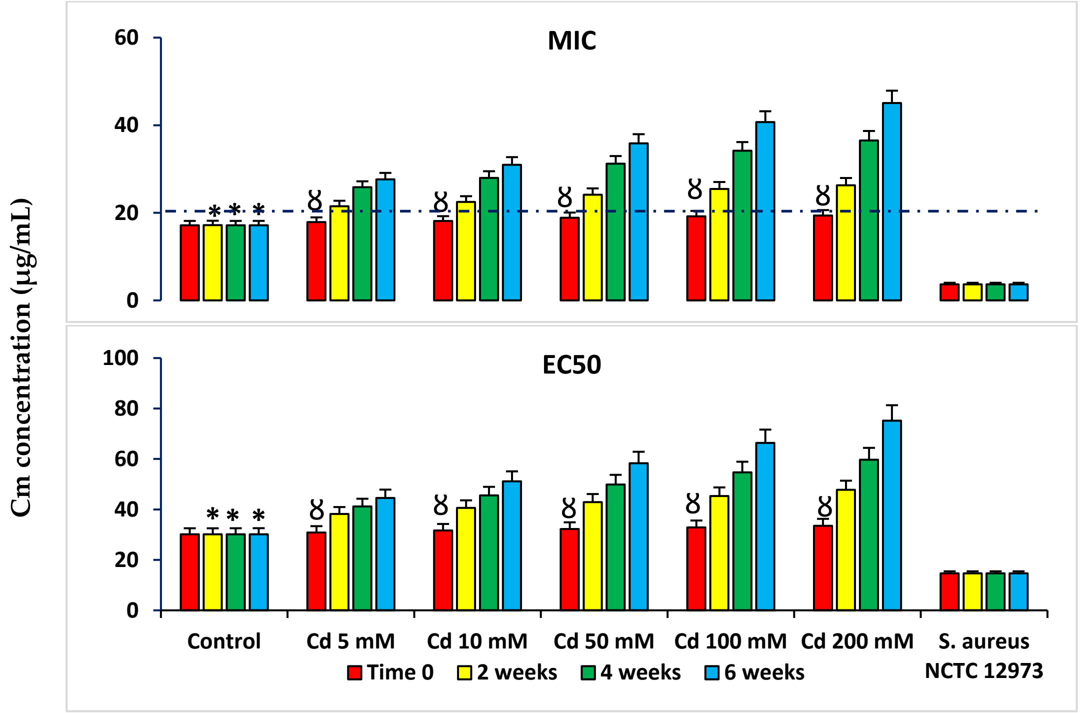

Figure S5. Mean MIC and EC50 values of PICT analysis with Cm for bacteria from Cd-spiked microcosms. *$p < 0.05$ compared to Cm MIC and EC50 values for bacteria from Cd-spiked microcosms at the same timepoint; 8$p < 0.05$ compared to Cm MIC and EC50 values for bacteria from Cd-spiked microcosms at 2, 4 and 6 week. The dash line defines AbR threshold for soil bacteria. *S.aureus* NCTC 12973 was a negative control.

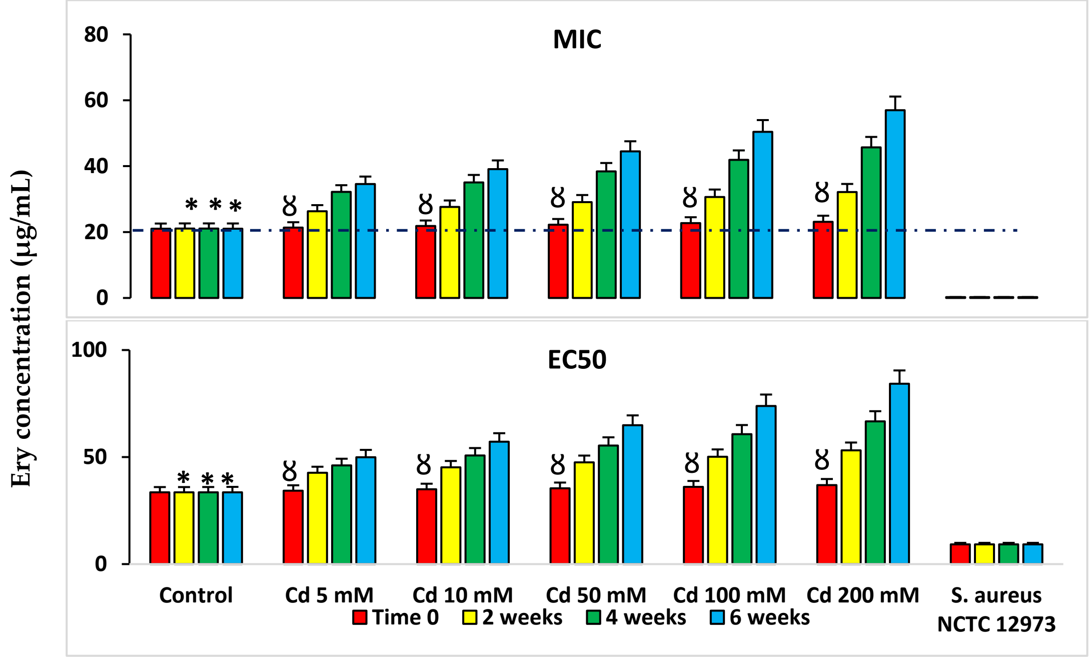

Figure S6. Mean MIC and EC50 values of PICT analysis with Ery for bacteria from Cd-spiked microcosms. *p < *0.05* compared to Ery MIC and EC50 values for bacteria from Cd-spiked microcosms at the same timepoint; 8p < *0.05* compared to Ery MIC and EC50 values for bacteria from Cd-spiked microcosms at 2, 4 and 6 week. The dash line defines AbR threshold for soil bacteria. *S.aureus* NCTC 12973 was a negative control.

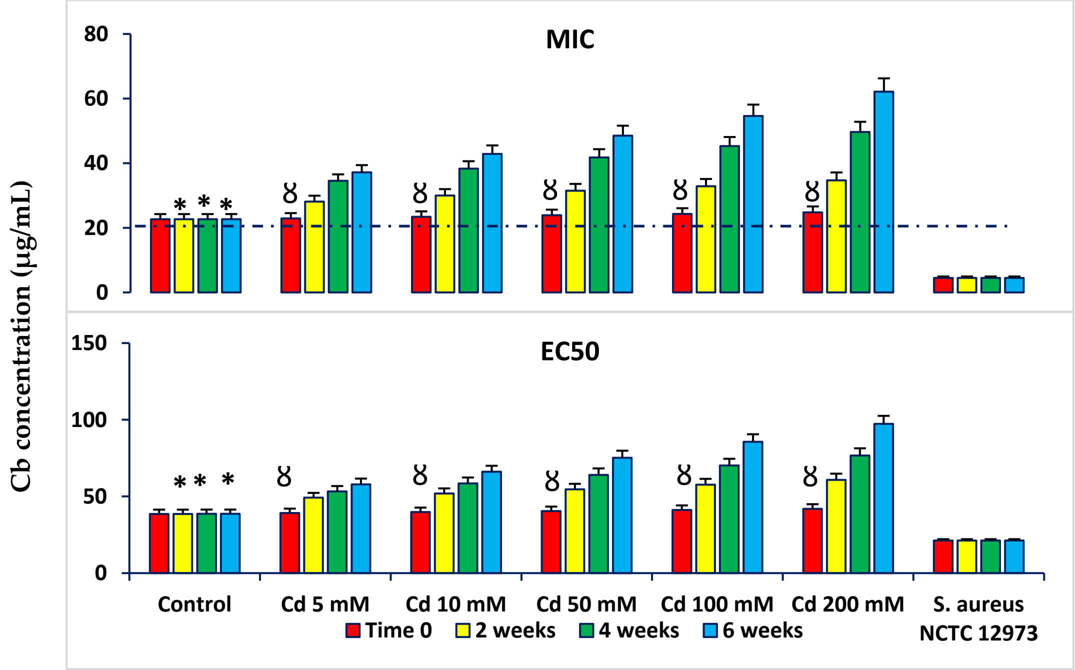

Figure S7. Mean MIC and EC50 values of PICT analysis with Cb for bacteria from Cd-spiked microcosms. *p < *0.05* compared to Cb MIC and EC50 values for bacteria from Cd-spiked microcosms at the same timepoint; 8p < *0.05* compared to Cb MIC and EC50 values for bacteria

from Cd-spiked microcosms at 2, 4 and 6 week. The dash line defines AbR threshold for soil bacteria. *S.aureus* NCTC 12973 was a negative control.

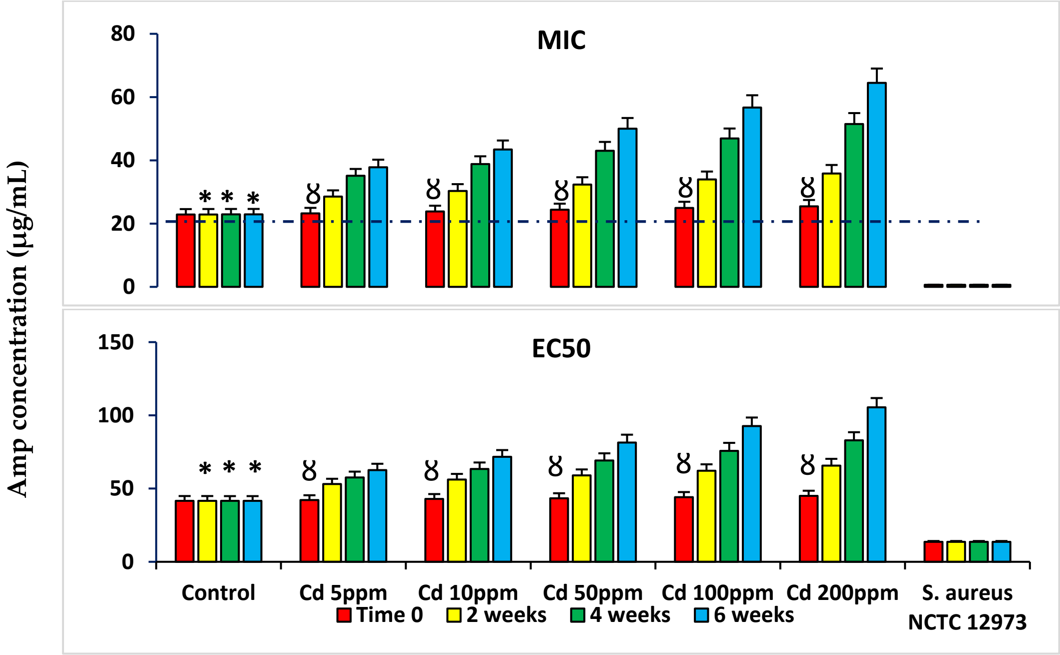

Figure S8. Mean MIC and EC50 values of PICT analysis with Amp for bacteria from Cd-spiked microcosms. *$p < 0.05$ compared to Amp MIC and EC50 values for bacteria from Cd-spiked microcosms at the same timepoint; 8$p < 0.05$ compared to Amp MIC and EC50 values for bacteria from Cd-spiked microcosms at 2, 4 and 6 week. The dash line defines AbR threshold for soil bacteria. *S.aureus* NCTC 12973 was a negative control.

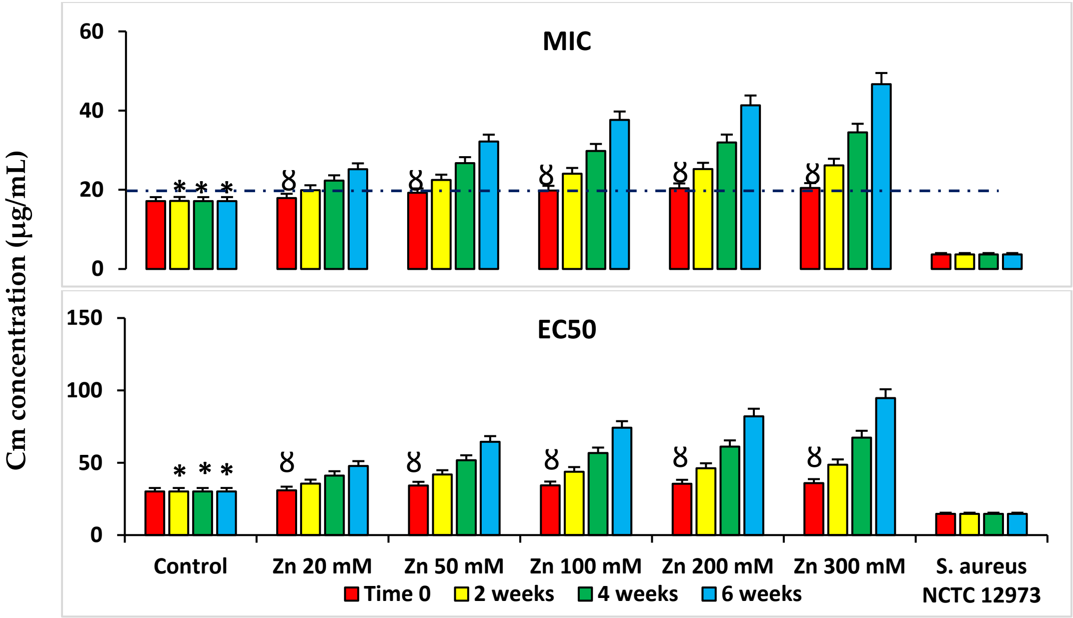

Figure S9. Mean MIC and EC50 values of PICT analysis with Cm for bacteria from Zn-spiked microcosms. *$p < 0.05$ compared to Cm MIC and EC50 values for bacteria from Zn-spiked microcosms at the same timepoint; 8$p < 0.05$ compared to Cm MIC and EC50 values for bacteria from Zn-spiked microcosms at 4 and 6 week. The dash line defines AbR threshold for soil bacteria. *S.aureus* NCTC 12973 was a negative control.

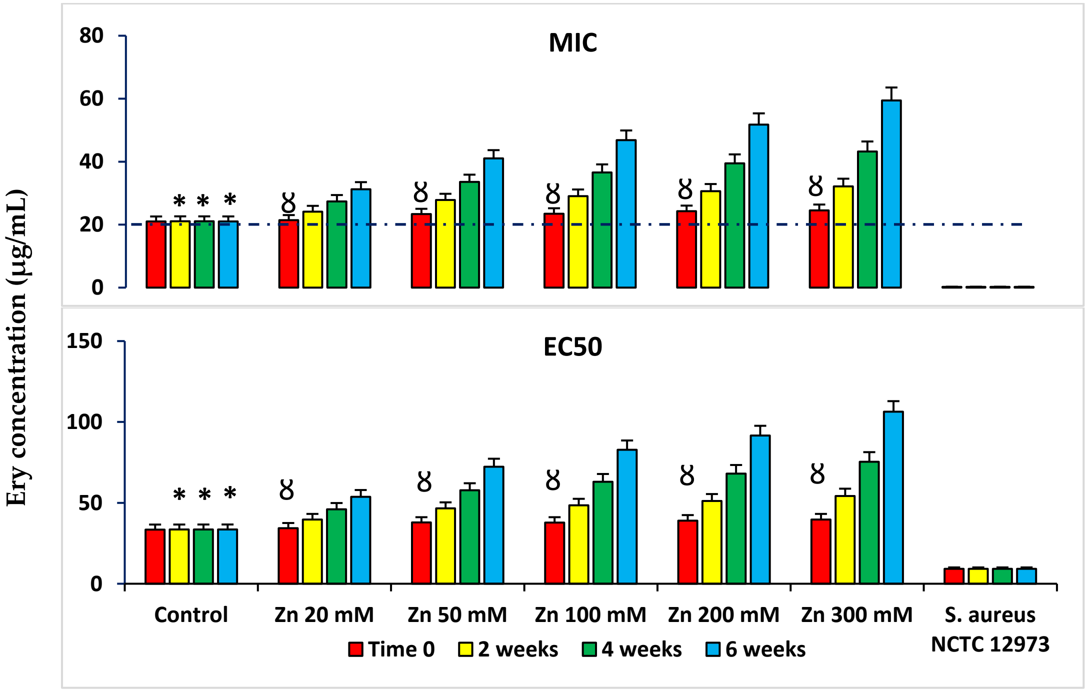

Figure S10. Mean MIC and EC50 values of PICT analysis with Ery for bacteria from Zn-spiked microcosms. *$p < 0.05$ compared to Ery MIC and EC50 values for bacteria from Zn-spiked microcosms at the same timepoint; 8$p < 0.05$ compared to Ery MIC and EC50 values for bacteria from Zn-spiked microcosms at 4 and 6 week. The dash line defines AbR threshold for soil bacteria. *S.aureus* NCTC 12973 was a negative control.

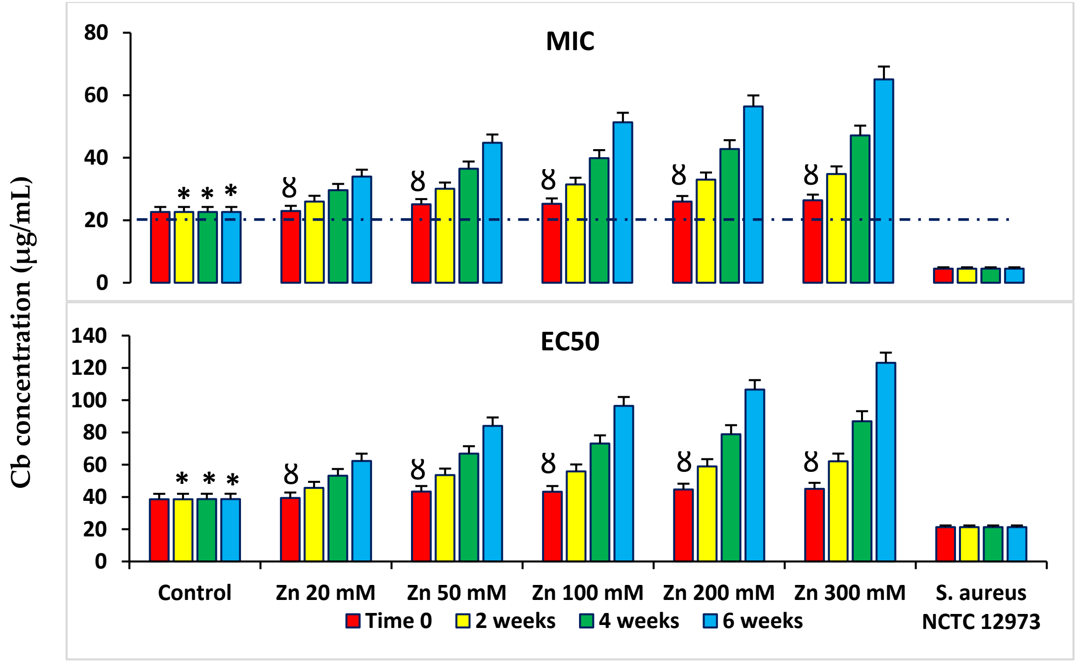

Figure S11. Mean MIC and EC50 values of PICT analysis with Cb for bacteria from Zn-spiked microcosms. *$p < 0.05$ compared to Cb MIC and EC50 values for bacteria from Zn-spiked microcosms at the same timepoint; 8$p < 0.05$ compared to Cb MIC and EC50 values for bacteria from Zn-spiked microcosms at 4 and 6 week. The dash line defines AbR threshold for soil bacteria. *S.aureus* NCTC 12973 was a negative control.

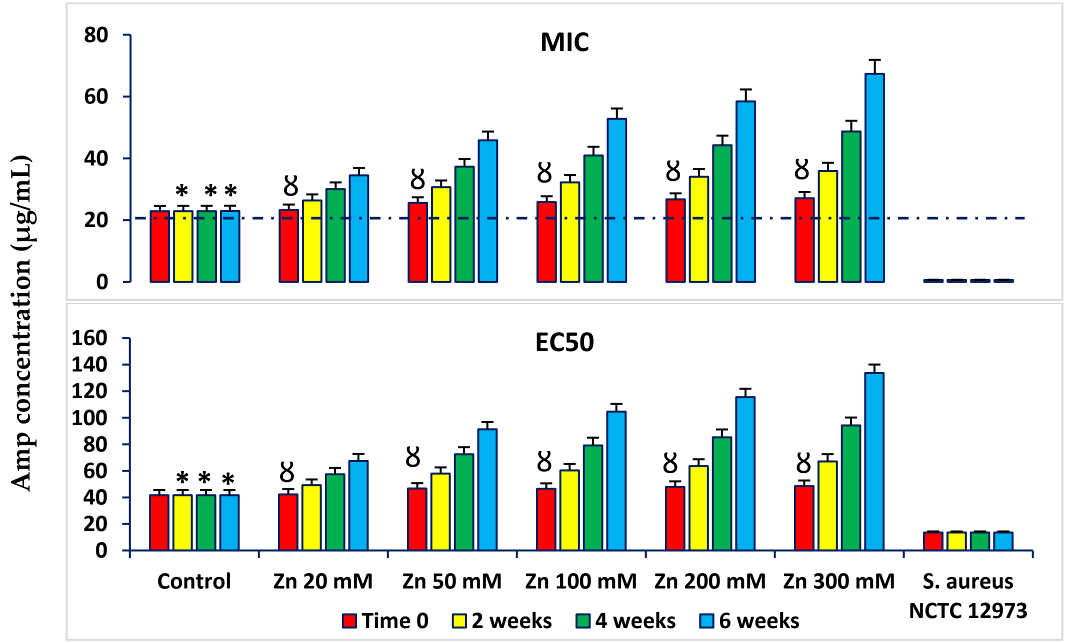

Figure S12. Mean MIC and EC50 values of PICT analysis with Amp for bacteria from Zn-spiked microcosms. *$p < 0.05$ compared to Amp MIC and EC50 values for bacteria from Zn-spiked microcosms at the same timepoint; 8$p < 0.05$ compared to Amp MIC and EC50 values for

bacteria from Zn-spiked microcosms at 4 and 6 week. The dash line defines AbR threshold for soil bacteria. *S.aureus* NCTC 12973 was a negative control.

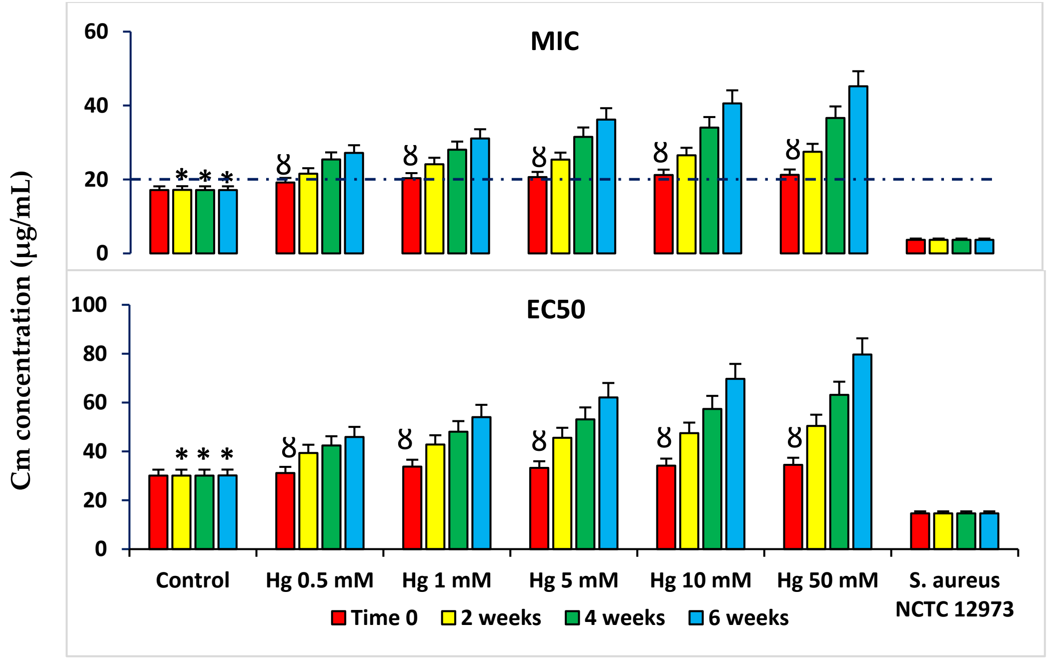

Figure S13. Mean MIC and EC50 values of PICT analysis with Cm for bacteria from Hg-spiked microcosms. *$p < 0.05$ compared to Cm MIC and EC50 values for bacteria from Hg-spiked microcosms at the same timepoint; 8$p < 0.05$ compared to Cm MIC and EC50 values for bacteria from Hg-spiked microcosms at 2, 4 and 6 week. The dash line defines AbR threshold for soil bacteria. *S.aureus* NCTC 12973 was a negative control.

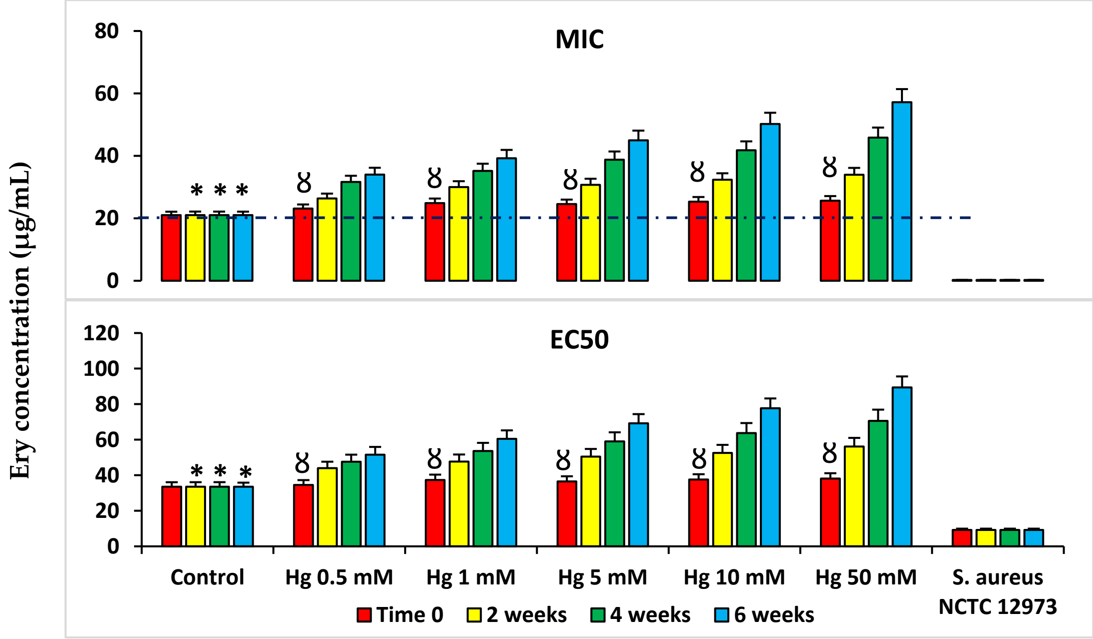

Figure S14. Mean MIC and EC50 values of PICT analysis with Ery for bacteria from Hg-spiked microcosms. *$p < 0.05$ compared to Ery MIC and EC50 values for bacteria from Hg-spiked microcosms at the same timepoint; 8$p < 0.05$ compared to Ery MIC and EC50 values for bacteria from Hg-spiked microcosms at 2, 4 and 6 week. The dash line defines AbR threshold for soil bacteria. *S.aureus* NCTC 12973 was a negative control.

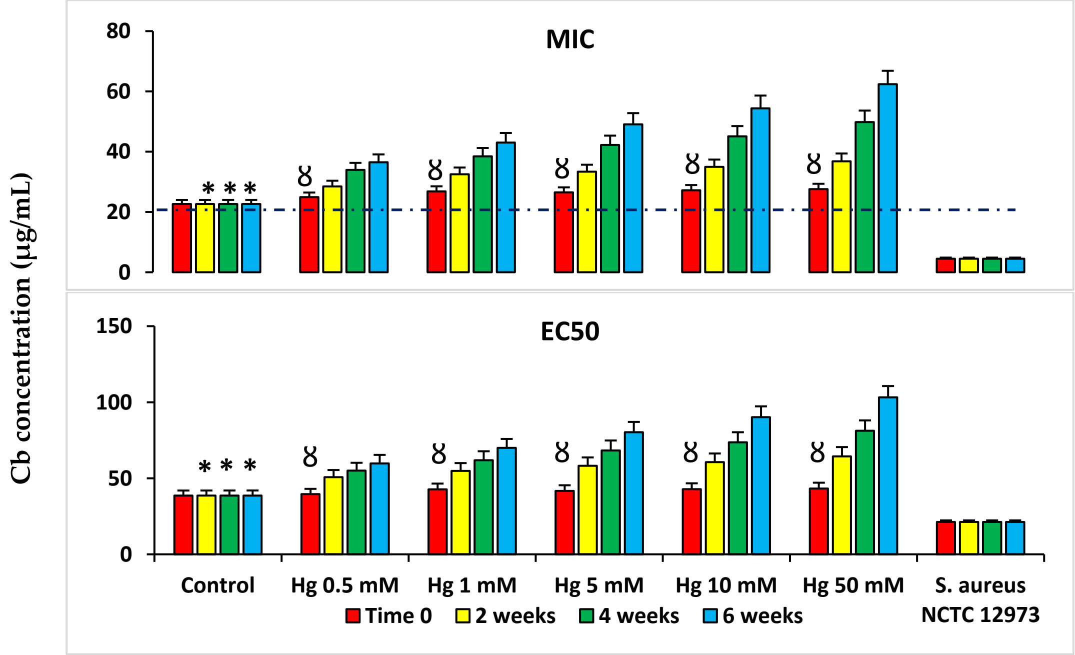

Figure S15. Mean MIC and EC50 values of PICT analysis with Cb for bacteria from Hg-spiked microcosms. *$p < 0.05$ compared to Cb MIC and EC50 values for bacteria from Hg-spiked microcosms at the same timepoint; 8$p < 0.05$ compared to Cb MIC and EC50 values for bacteria

from Hg-spiked microcosms at 2, 4 and 6 week. The dash line defines AbR threshold for soil bacteria. *S.aureus* NCTC 12973 was a negative control.

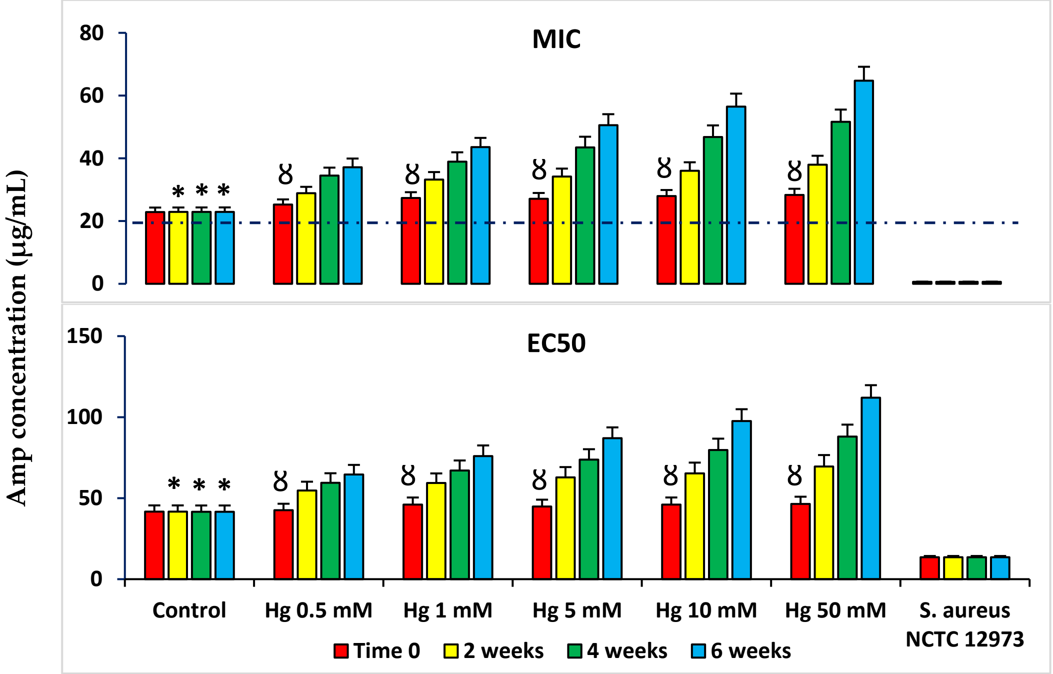

Figure S16. Mean MIC and EC50 values of PICT analysis with Amp for bacteria from Hg-spiked microcosms. **p* < *0.05* compared to Amp MIC and EC50 values for bacteria from Hg-spiked microcosms at the same timepoint; 8*p* < *0.05* compared to Amp MIC and EC50 values for bacteria from Hg-spiked microcosms at 2, 4 and 6 week. The dash line defines AbR threshold for soil bacteria. *S.aureus* NCTC 12973 was a negative control.

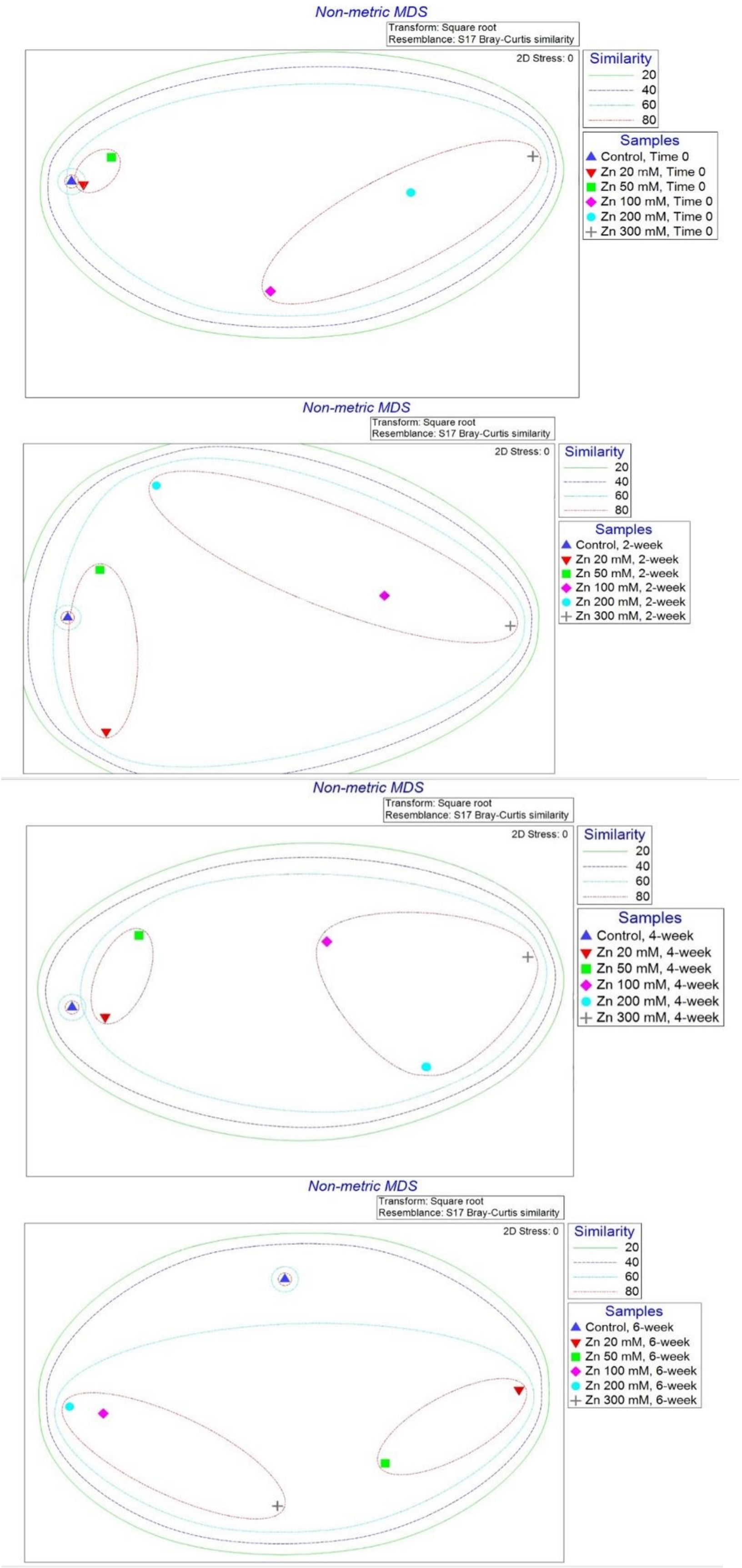

Figure S17. NMS analysis plot of TRFLP relative peak height for the Zn-spiked microcosm soil's bacterial communities' data, using the Bray-Curtis similarity index. Significant difference ($p < 0.05$) between the clusters specified with >60% of similarity compared to control microcosms.

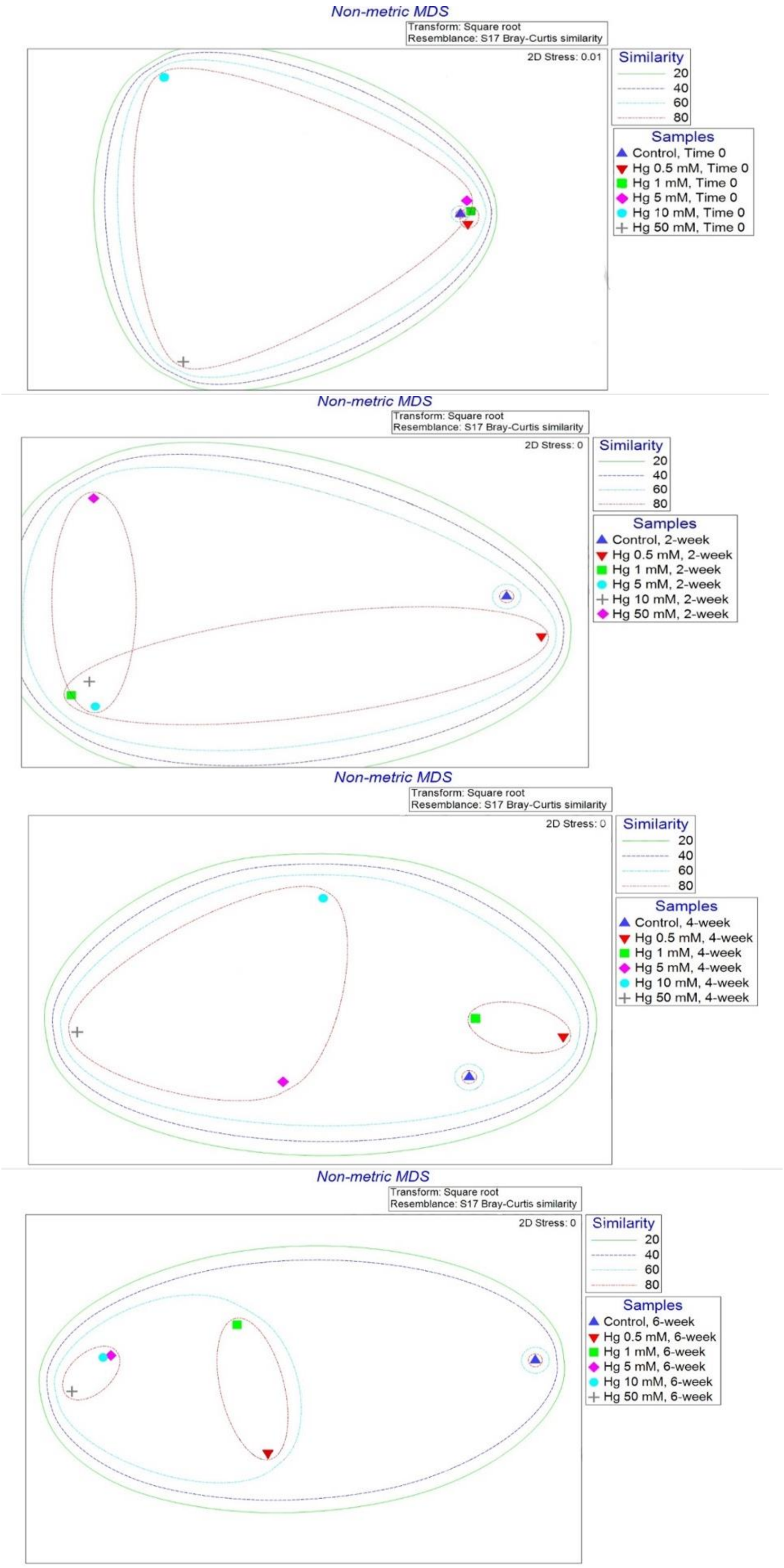

Figure S18. NMS analysis plot of TRFLP relative peak height for the Hg-spiked microcosm soil's bacterial communities' data, using the Bray-Curtis similarity index. Significant difference ($p < 0.05$) between the clusters specified with >60% similarity compared to control microcosm.
